# MCPNet: a parallel maximum capacity-based genome-scale gene network construction framework

**DOI:** 10.1093/bioinformatics/btad373

**Published:** 2023-06-08

**Authors:** Tony C Pan, Sriram P Chockalingam, Maneesha Aluru, Srinivas Aluru

**Affiliations:** Department of Biomedical Informatics, Emory University, Woodruff Memorial Research Building 101 Woodruff Circle, 4th Floor East, Atlanta, GA 30322, United States; Institute for Data Engineering and Science, Georgia Institute of Technology, 756 W Peachtree St NW, 12th Floor, Atlanta, GA 30332, United States; Institute for Data Engineering and Science, Georgia Institute of Technology, 756 W Peachtree St NW, 12th Floor, Atlanta, GA 30332, United States; School of Biological Sciences, Georgia Institute of Technology, 310 Ferst Dr NW, Atlanta, GA 30332, United States; Institute for Data Engineering and Science, Georgia Institute of Technology, 756 W Peachtree St NW, 12th Floor, Atlanta, GA 30332, United States; School of Computational Science and Engineering, Georgia Institute of Technology, 756 W Peachtree St NW, 13th Floor, Atlanta, GA 30332, United States

## Abstract

**Motivation:**

Gene network reconstruction from gene expression profiles is a compute- and data-intensive problem. Numerous methods based on diverse approaches including mutual information, random forests, Bayesian networks, correlation measures, as well as their transforms and filters such as data processing inequality, have been proposed. However, an effective gene network reconstruction method that performs well in all three aspects of computational efficiency, data size scalability, and output quality remains elusive. Simple techniques such as Pearson correlation are fast to compute but ignore indirect interactions, while more robust methods such as Bayesian networks are prohibitively time consuming to apply to tens of thousands of genes.

**Results:**

We developed maximum capacity path (MCP) score, a novel maximum-capacity-path-based metric to quantify the relative strengths of direct and indirect gene–gene interactions. We further present MCPNet, an efficient, parallelized gene network reconstruction software based on MCP score, to reverse engineer networks in unsupervised and ensemble manners. Using synthetic and real *Saccharomyces cervisiae* datasets as well as real *Arabidopsis thaliana* datasets, we demonstrate that MCPNet produces better quality networks as measured by AUPRC, is significantly faster than all other gene network reconstruction software, and also scales well to tens of thousands of genes and hundreds of CPU cores. Thus, MCPNet represents a new gene network reconstruction tool that simultaneously achieves quality, performance, and scalability requirements.

**Availability and implementation:**

Source code freely available for download at https://doi.org/10.5281/zenodo.6499747 and https://github.com/AluruLab/MCPNet, implemented in C++ and supported on Linux.

## 1 Introduction

Gene network inference from high-throughput gene expression data is a compute intensive task. Although several different inference algorithms based on Bayesian networks (Hartemink 2005), mutual information (MI) theory (Faith *et al.* 2007, Aluru *et al.* 2013) Pearson correlation (Langfelder and Horvath 2008), regression (Bonneau *et al.* 2006, Huynh-Thu *et al.* 2010), and random forest (Aibar *et al.* 2017) have been developed over the past two decades, scalability remains a critical challenge when working with tens of thousands of genes and/or observations. Aluru *et al.* (2022) found that 9 of the 15 methods analysed failed to infer networks from a dataset with ∼18 000 genes. Furthermore, of the six that succeeded, four required between 4 and 49 days to complete. To construct large networks with tens of thousands of genes in reasonable time, scalable algorithms and efficient parallel implementations are necessary. Arboreto (Moerman *et al.* 2019) recently introduced distributed computing capability to construct random forest-based transcription factor (TF)-target gene regulatory networks (GRNs); however, this is still not scalable for large networks. TINGe (Zola *et al.* 2010), using a parallel MI-based approach can construct large genome-scale gene networks in a significantly shorter amount of time.

Past surveys conducted with established benchmark data suggest that Bayesian and MI-based methods are among the best performing (Marbach *et al.* 2012, Lachmann *et al.* 2016, Aluru *et al.* 2022) with respect to the quality and accuracy of inferred interactions. However, unlike Bayesian networks, MI-based methods are more amenable to large scale network reconstruction (Chockalingam *et al.* 2017). One key caveat though is that these methods require post-processing such as Stouffer Transform in CLR (Faith *et al.* 2007) or MI value filtering based on *P*-values and data processing inequality (DPI) measures in ARACNe-adaptive partitioning (AP) (Lachmann *et al.* 2016) and TINGe. Such filtering complicates network evaluation by standard metrics such as the area under the precision-recall curve (AUPRC) as it introduces discontinuities in the value range. Additionally, DPI requires a user-supplied tolerance parameter that is challenging to determine a priori. As DPI reflects the information transmission capacity, it is a special case of the maximum capacity path (MCP) problem in graph theory, which has previously found applications in network routing (Pollack 1960), image compositing (Fernandez *et al.* 1998), and metabolic pathway analysis (Ullah *et al.* 2009). Formulated as such, the tolerance parameter is no longer required.

In this paper, we present a novel gene network reconstruction approach based on MCP to characterize and compare indirect gene interactions to identify significant gene–gene interactions or TF–target relationships without thresholding or user-specified parameters. Our unsupervised approach examines all indirect paths between two genes to compute the maximum indirect interaction capacity and allows efficient investigation of paths of arbitrary lengths. Using the same framework, we also established an ensemble method that combines interactions from multiple path lengths using optimized weights identified with partial groundtruth. We further created an efficient parallel implementation of the algorithm, called MCPNet, that can scale to hundreds of cores. We evaluated performance of our unsupervised and ensemble approaches using MI as the direct interaction measure and paths with length up to four for both gene–gene and TF–target interactions and demonstrate that our method delivers higher network quality and superior computational performance when compared to other state-of-the-art gene network reconstruction software.

## 2 Materials and methods

A gene expression dataset with samples *S* is formulated as a matrix *P* with |V| rows and |S| columns, each row corresponding to expression levels for a gene, and each column corresponding to the gene expression profile of a sample in *S*. Here, we reconstruct and examine two different types of gene networks—gene co-expression networks (GCN) and GRN. GCN is defined as an undirected weighted graph G=〈V,E,W〉, where $V=\{v_{1}\ldots v_{N}\}$ is the set of *N* genes, $E=\{(v_{i},v_{j})|v_{i},v_{j}\in V\}$ is the set of edges representing interacting genes, and *W* is a matrix of size *N *×* N*, where the entry $W_{ij}$ is an edge weight that quantifies the interaction strength of *v_i_* with *v_j_*. GRN is directed in contrast to a GCN, and can be defined as G=〈U,V,E,W〉, where $U=\{u_{1}\ldots u_{M}\}$ represent the regulators, *V* is the set of *N* target genes. The edge set is $E=\{(u_{i},v_{j})|u_{i}\in U,v_{j}\in V\}$, and $W_{ij}$ contains the weights corresponding to the edges in *E*. Since a GRN is directed, *W* is rectangular.

Different simplifying mathematical models have been adopted to compute the edge weights. Correlation measures such as Pearson computes $W_{ij}$ from the expression profiles of genes *v_i_* and *v_j_*. Correlation measures are commutative, $W_{ij}=W_{ji}$, thus the square weight matrix of GCN is symmetric but may contain negative elements. In the Arboreto model (Huynh-Thu *et al.* 2010), $W_{ij}$ indicates the degree to which the expression of gene *v_i_* can predict *v_j_*’s gene expression, thus GCN *W* is non-negative but may not be symmetric. An alternative to correlation is MI, where the expression profiles for genes $v_{i},v_{j}\in V$, rows *i* and *j* in matrix *P* are modeled as random variables *X_i_* and *X_j_*, from which MI is estimated. Since MI is non-negative and commutative, GCN *W* is symmetric and positive semi-definite. As these measures compute pair-wise interaction strengths, the weight matrix *W* of a GRN can be computed element-wise in the same way as *W* for a GCN.

Our proposed method transforms the input edge weight matrix *W* to produce a new MCP score that accounts for indirect interaction strengths and can leverage different interaction strength measures while preserving the directionality of the measures. In this paper, MI is used to demonstrate the performance of our proposed method, though our method is agnostic of the semantics of the *W* matrix or its symmetry and thus is suited for both undirected GCN and directed GRN reconstruction. For *W* with negative values, our proposed method can use the magnitude, i.e. absolute value, of the interaction strength instead.

### 2.1 MCP score

We define an indirect interaction between two genes as one that is mediated through one or more intermediary genes and can be modeled as a length-*L* path $\left(v_{s},\ldots ,v_{i_{r}},\ldots ,v_{t}\right)$ in *G*, where *v_s_* and *v_t_* are source and target genes, $v_{i_{r}}$ is an intermediary gene in *V* indexed by $i_{r}\in \{1\ldots |V|\}$, and r∈{1…L−1} indicates position along the path. Indirect biological interactions are well-known in eukaryotic organisms—e.g. the gene networks of biochemical, cellular, and signal transduction pathways where a change in the structure or function of a gene/protein can have indirect effects on pathway genes that are not adjacent to each other through regulatory positive or negative feedback (Harris and Levine 2005, Lu *et al.* 2007, Mitrophanov and Groisman 2008, Itzhack *et al.* 2013, Vermeirssen *et al.* 2014, Rittschof and Robinson 2016, Cowen *et al.* 2017). Reconstructing the network from gene expression profiles is guided by two goals: identifying strong direct interactions between gene pairs, and simplifying the network by removing direct interactions made redundant by strong indirect interactions. Our proposed method seeks to accomplish both by first identifying the strongest indirect interactions between two genes, then compare it to the direct interaction strength.

**Figure 1.**
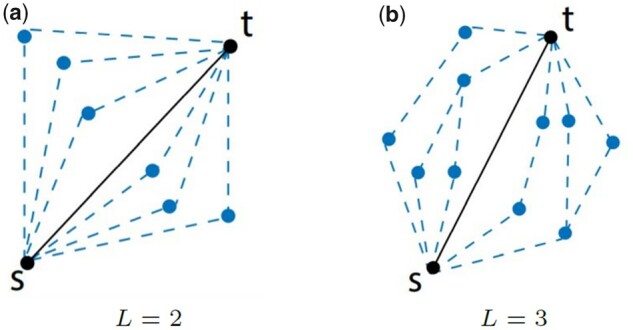
Computing $\eta_{st}^{2}$ and $\eta_{st}^{3}$ in a network requires exploring different paths with one and two intermediate vertices, respectively, as shown above.

The maximum indirect interaction strength is determined by solving the MCP problem. Figure 1 illustrates different length-2 and length-3 paths between genes *v_s_* and *v_t_* as dashed lines and their direct interactions as solid lines. For each path, the minimum edge weight is its maximum capacity. Among all the possible length-*L* paths between *v_s_* and *v_t_* in *G*, the MCP is the path with the highest minimum edge weight. We use $\eta_{st}^{L}$ to denote the capacity of such a path between *v_s_* and *v_t_*, and refer to it as *L-path Capacity* or *Path Capacity*. Formally,


(1) $$\eta_{st}^{L}=\max_{i_{1},\ldots ,{\;i}_{L-1}\in \{1\ldots |V|\}}(\min(W_{si_{1}},W_{i_{1}i_{2}},\ldots ,W_{i_{L-1}t}))$$

The maximum operation finds a combination of indices $i_{1},\ldots ,{\;i}_{L-1}$ with the maximum path capacity, with each index having range {1…|V|}. Multiple indices may refer to the same gene, thus forming a cycle in the *L*-path, representing, e.g. feedback loops for gene regulation. In subsequent sections, the range {1…|V|} for the indices is implied in the maximum operation.

Once computed, $\eta_{st}^{L}$ is compared to the direct interaction strength $W_{st}$. We define the *L*-path MCP score between the genes *v_s_* and *v_t_*, denoted as $\rho_{st}^{l}$, as the following ratio:


(2) $$\rho_{st}^{L}=W_{st}/\eta_{st}^{L}$$

We use both the terms *L-path MCP Score* and *MCP Score* to refer to $\rho_{st}^{L}$. The output of our proposed method is denoted $R^{L}$, a matrix of all the $\rho_{st}^{L}$’s for every pair of *s* and *t*. As a ratio of the edge weight to the path capacity, we expect $\rho_{st}^{L}$ to be an indicator of the interaction strength of genes *v_s_* and *v_t_* while factoring in the effect of other interactions in the *L*-neighborhood of *v_s_* and *v_t_*. Strong direct interactions relative to indirect ones suggest that the edge should be kept in the network. We note that the DPI-based approach used by ARACNe-AP and TINGe can be modeled as a specialization of the *L*-path MCP score, as shown in Supplementary Section S1.1. Whereas ARACNe-AP and TINGe uses a DPI threshold to filter the MI matrix thus removing edges representing weak direct interactions compared to their indirect counterparts, our approach quantifies the relative strengths of the direct and indirect interactions and allows for post-processing according to application needs, e.g. thresholding to retain relatively strong direct interactions by examining the precision-recall curve.

### 2.2 MCP score algorithm

The 2-path capacity $\eta_{st}^{2}=\underset{i}{\max}(\min(W_{si},W_{it}))$ can be computed explicitly by enumerating all possible length-2 paths between *v_s_* and *v_t_*. Algorithm 1 shows the pseudo-code for this approach. The 2-path MCP score defined in Section 2.1, $\rho_{st}^{2}$, can be calculated for the complete network directly by first computing $\eta_{st}^{2}$ for a pair of genes via Algorithm 1, iterating over the two gene expression profiles in O(|V|) time. The algorithm has a run-time complexity of $O(|V{|}^{3})$ as it iterates over all elements of a |V|×|V| matrix (Algorithm 2).

Algorithm 1: Path Capacity Kernel for Path Length 2

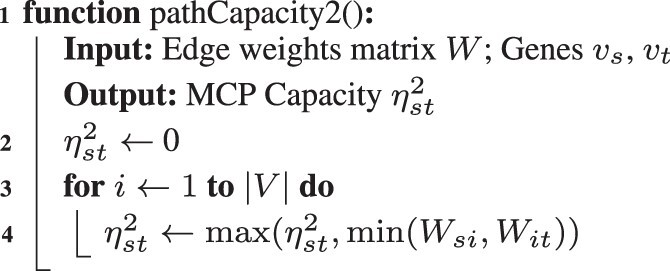

Algorithm 2: MCP Score

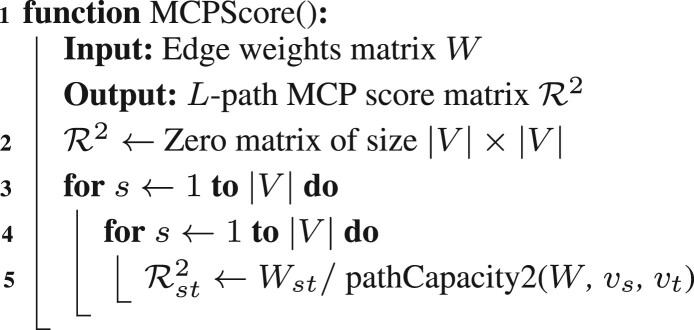

Algorithm 2 applies for *L*-path MCP scores for any arbitrary *L*. Naive extension of Algorithm 1 to longer range interactions is exponential in computational complexity, however, as an increase of *L* by 1 increases computation by |V|-fold. For an indirect interaction of length *L*, the naive $\eta_{st}^{L}$ computation complexity is $O(|V{|}^{L-1})$, leading to $O(|V{|}^{l+1})$ to compute the scores for all gene–gene pairs.

Pollack (1960) showed that the maximum capacity $\eta_{st}^{L}$ of all length-*L* paths between two vertices in a graph can be computed efficiently via recursive path bisection. Formally, where h=⌊L/2⌋. The length-2 realization, $\eta_{st}^{2}$, forms the base case for the recursion. This partitioning leverages the associative properties of the maximum and minimum operations. Let $c_{si_{h}}=\min(W_{si_{1}},\ldots ,W_{i_{h-1}i_{h}})$ and $c_{i_{h}t}=\min(W_{i_{h}i_{h+1}},\ldots ,W_{i_{L-1}t})$. Equation (1) can then be rewritten as


(3) $$\eta_{st}^{L}=\underset{i_{h}}{\max}(\min(\eta_{si_{h}}^{h},\eta_{i_{h}t}^{L-h}))$$

$$\begin{array}{c} \eta_{st}^{L}=\underset{i_{1},\ldots ,i_{L-1}}{\max}(\min(c_{si_{h}},c_{i_{h}t}))\\ =\underset{i_{h}}{\max}(\underset{i_{1},\ldots ,i_{h-1}}{\max}(\underset{i_{h+1},\ldots ,i_{L-1}}{\max}(\min(c_{si_{h}},c_{i_{h}t})))) \end{array}$$

An *L*-path does not require unique genes on the path. This implies that $c_{si_{h}}$ does not depend on $i_{h+1}\ldots i_{L-1}$, and $c_{si_{h}}$ is effectively a constant with respect to the $\underset{i_{h+1},\ldots ,i_{L-1}}{\max}()$ operation. Since the maximum and minimum operators are distributive over each other with respect to a constant, i.e. max(min(z,x),min(z,y))=min(z,max(x,y)),


$$\begin{array}{c} \eta_{st}^{L}=\underset{i_{h}}{\max}(\underset{i_{1},\ldots ,i_{h-1}}{\max}(\min(c_{si_{h}},\underset{i_{h+1},\ldots ,i_{L-1}}{\max}(c_{i_{h}t}))))\\ =\underset{i_{h}}{\max}(\underset{i_{1},\ldots ,i_{h-1}}{\max}(\min(c_{si_{h}},\eta_{i_{h}t}^{L-h}))) \end{array}$$

Similarly $\eta_{i_{h}t}^{L-h}$ is constant with respect to $i_{1}\ldots i_{h-1}$. Applying the identity again leads to Equation (3).

Algorithm 3: Recursive Path Capacity Kernel for Length L

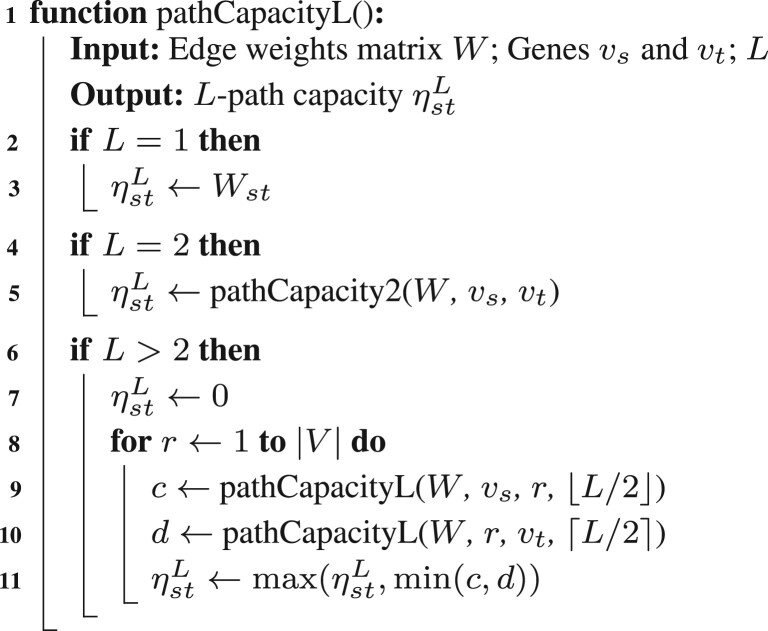

The recursive path bisection allows *L*-length MCP scores to be computed in $O(|V|{\;\text{log}\;}_{2}L)$ for a single gene–gene pair, and the *L*-path capacity for all gene pairs to be computed in $O(|V{|}^{3}{\;\text{log}\;}_{2}L)$ time. A realization of Equation (3) is shown in Algorithm 3. For a gene pair (*v_s_*, *v_t_*), the *η* values for half-length indirect interactions between *v_s_* and *v_t_* with all possible intermediary genes $v_{r}\in V$ are computed. The *η* value for (*v_s_*, *v_t_*) is then computed following the MCP problem solution as the two-path capacity case.

### 2.3 Parallel implementation

MCPNet pipeline consists of a sequence of functions. We first compute the MI between pairs of genes, using the gene expression profile matrix as input. The resulting MI matrix was then transformed to MCP score via the algorithm described in the sections above. Our implementations of Algorithms 1–3 are optimized and parallelized for multi-core and multi-node systems.

First, we note that Algorithm 2 has data access pattern identical to matrix–matrix multiplication, with Algorithm 1 replacing vector dot product as the computational kernel. Indeed, fast matrix multiply algorithms have been applied to the MCP problem (Vassilevska *et al.* 2007, Duan and Pettie 2009) to achieve sub-cubic run-time. For simplicity and ease of parallelization, we compute each *η* directly in O(|V|) time. In contrast to the *dominant product* algorithm by Vassilevska *et al.* (2007) that requires random memory access, our algorithm incurs only sequential memory access and therefore is cache-friendly and can leverage hardware memory prefetching.

Since each element can be computed independently from the other elements of the output matrix, a simple parallelization scheme is sufficient. The output matrix is partitioned into 8 × 8 tiles and tiles are assigned to the compute nodes and CPUs. Use of tiles improves cache reuse as the eight rows and eight columns of the input matrices needed to compute one tile are likely to remain in cache memory during computation.

**Figure 2.**
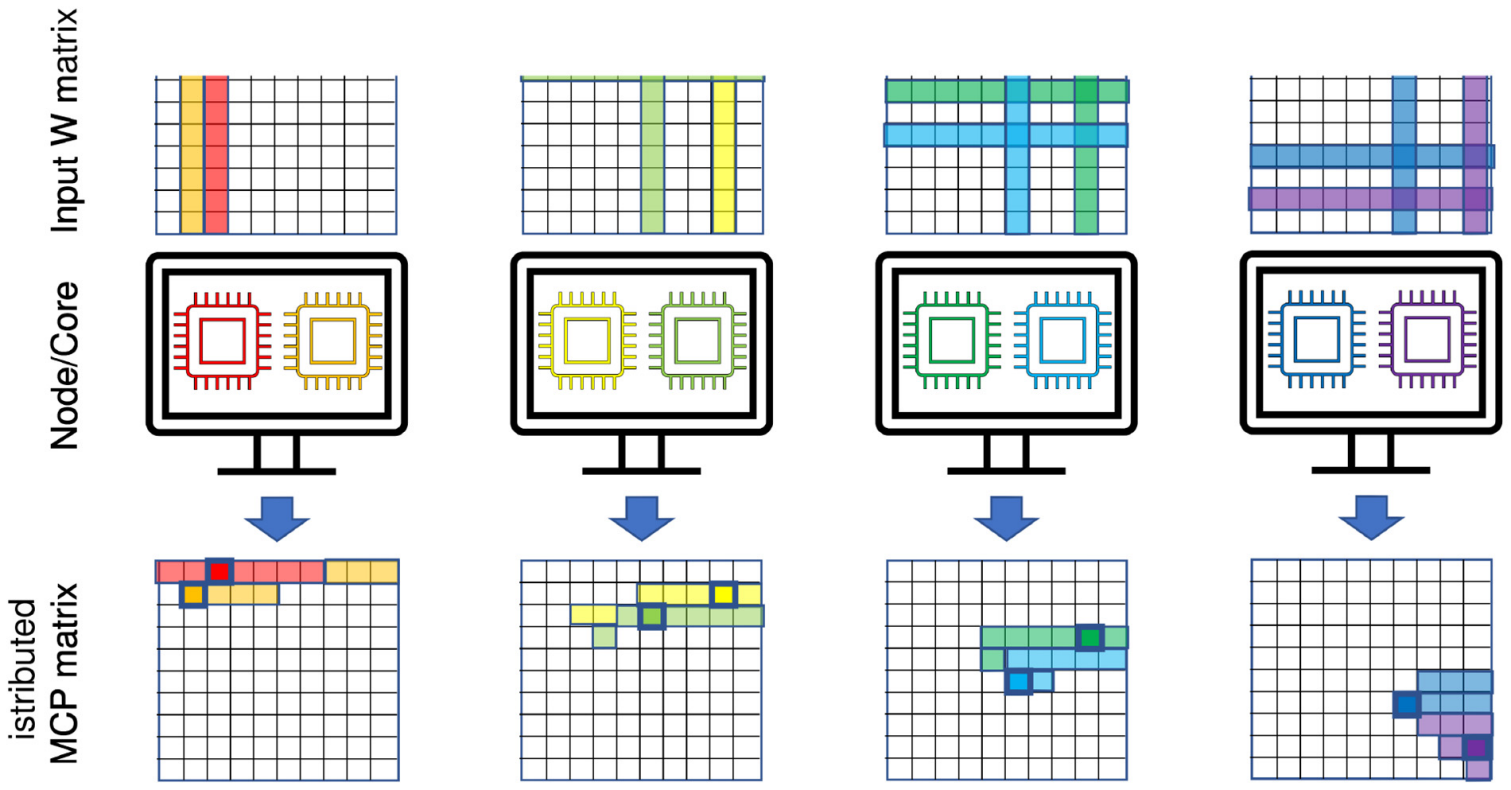
Paralellization scheme for the MCP score algorithm. The input W matrix is replicated to all nodes and shared between cores of a node. Each core (colored) computes some tiles (light shading in the MCP matrix) in the MCP matrix thus the output matrix is distributed across the compute nodes. For each MCP tile being computed (solid square with thick border in the MCP matrix), the required rows and columns are shaded in the W matrix according to the core color.

To ensure that the required input matrix row and column to compute one $\eta_{st}^{2}$ value are in local memory, the MI matrix is replicated on each compute node, and shared between cores of the same node. The MI computation is similarly organized across all nodes, with the gene expression profile matrix replicated and the MI matrix tiles partitioned across cores. One round of all-to-all personalized communication via message passing interface reconstructs the MI or MCP score matrices on all compute nodes. The input replication reduces communication during computation. The overall parallelization approach is shown in Figure 2.

Finally, the computation to produce $\eta_{st}^{2}$ is readily vectorized using element-wise minimum and maximum operators. For reconstruction where the input weight matrix *W* is symmetric, the MCP score matrix is symmetric as well thus half of the computation can be avoided.

### 2.4 Ensemble multipath MCP scores

MCP scores as defined in Section 2.1 are for a fixed path length. To account for indirect interactions of multiple lengths, we consider a linear combination of the *η* values in computing an ensemble DPI score: where *a*_2_, *a*_3_, …, *a_L_* are coefficients for the different indirect interaction path lengths, with the standard constraint that the weights add to 1.0.


(4) $$M_{st}^{L}=W_{st}/(a_{2}\times \eta_{st}^{2}+a_{3}\times \eta_{st}^{3}+\cdots +a_{L}\times \eta_{st}^{L})$$

The optimal ensemble parameters are generally challenging to determine. We propose to use available partial groundtruth to help optimize the ensemble parameters. For large gene networks, there are known and experimentally validated gene–gene and TF–target interactions. Using the known interactions as partial groundtruth for optimizing the ensemble parameters, the proposed ensemble approach promises to improve predictions of unknown interactions in the remainder of the network.

For the ensemble multi-path MCP score, we compute a weighted average of the 2-, 3-, and 4-path capacities by iterating over combinations of coefficients at a default interval of 0.1 for each coefficient. The AUPRC of the resulting networks are computed based on known interactions. The coefficient combination with the maximum AUPRC is used as the optimal combination for the ensemble. The ensemble approach allows the network inferring process to adjust to the dataset available, the organism of interest, and the available partial groundtruth.

## 3 Evaluation methodology

We evaluate performance of MCPNet using both simulated and real datasets, and compare it with seven other popular gene network reconstruction software—four MI-based methods [ARACNe-AP (Lachmann *et al.* 2016), CLR (Faith *et al.* 2007), MRNET(Meyer *et al.* 2008), TINGe (Aluru *et al.* 2013)], a Pearson correlation-based method (WGCNA; Langfelder and Horvath 2008), a random forest-based method (Arboreto; Aibar *et al.* 2017), and a regression-based method (Inferelator; Bonneau *et al.* 2006). Arboreto and Inferelator are ensemble methods, in contrast to the other methods referenced above which are standalone. Each method evaluated is executed with its default parameters, or published parameters for the target dataset (Tchourine *et al.* 2018). We applied the standard statistical measure AUPRC for assessing the inferred networks. AUPRC is the area under the precision-recall curve, and is recommended in cases where there is an imbalance of positive and negative edges in the underlying network (Davis and Goadrich 2006). Using AUPRC for both ensemble optimization and final network evaluation leads to the ensemble network having the maximal AUPRC for the given partial groundtruth. However, when different groundtruth are used to parameterize the AUPRC function, the generalization performance of the MCPNet ensemble method can be assessed as demonstrated in Section 4.4.

For *in silico* evaluations, we used NetBenchmark (Bellot *et al.* 2015), an R Bioconductor package that internally employs the GeneNetWeaver simulator (Schaffter *et al.* 2011) to generate synthetic yeast datasets. NetBenchmark uses 5196 real interactions from the Yeast Transcriptional Regulatory Network (Balaji *et al.* 2006) and employs non-linear ordinary differential expression simulation to produce 2000 observations for 2000 genes. Noise is then injected at five different levels in ten replicas to create 51 total datasets. The 5196 real interactions are used as true positives in the groundtruth, while the remaining 1 993 804 gene-pairs are considered as true negatives. The groundtruth for the simulated, as well as real Yeast and *Arabidopsis thaliana* are majority TF–target interactions and are used for both GCN and GRN evaluations. For synthetic TF–target network reconstruction, 187 TFs from Inferelator source repository (Castro *et al.* 2019) were included.

To determine accuracy and scalability of gene network reconstruction with real-world data, we used gene expression data from two different organisms—*S. cerevisiae* and *A. thaliana*. The *S. cerevisiae* (yeast) dataset is a compilation of multiple yeast RNA-seq expression studies, and contains 2577 observations and 5716 genes (Tchourine *et al.* 2018). To determine the quality of the real-world yeast networks, we adopted the groundtruth from Castro *et al.* (2019), consisting of a matrix with 97 TFs and 956 targets. The 1360 non-zero entries are considered as true positives, while the remaining 91 372 are true negatives. Castro *et al.* generated the matrix by combining TF-binding and TF-knockout data collected by Tchourine *et al.* (2018) from YEASTRACT (Teixeira *et al.* 2006) and SGD (Costanzo *et al.* 2014) databases, and TF–target interactions derived from chromatin accessibility data (Boyle *et al.* 2008, Buenrostro *et al.* 2013) and TF binding sites (Weirauch *et al.* 2014) by associating TFs with the closest downstream genes. For real yeast TF–target network reconstruction, the 563 TFs from the Inferelator source repository (Castro *et al.* 2019) were included.

The *A. thaliana* microarray data was downloaded from public repositories (Aluru *et al.* 2022), and processed according to (Chockalingam *et al.* 2016). Data from six different tissues/conditions with varying sizes of gene expression datasets (Table 1) were used for gene network reconstruction. Microarray data was used as it was readily available from our previous studies, and it demonstrated the applicability of the MCPNet methods to both RNAseq and microarray data. *Arabidopsis thaliana* network(s) were evaluated using interactions from the following two known networks as groundtruth: (i) Arabidopsis Transcriptional Regulatory Map (ATRM) constructed from *a priori* biological knowledge by Jin *et al.* (2015), which includes 1359 TF–target interactions; (ii) 295 highest confidence TF–target interactions with scores greater than 9.5 selected from the 6863N-response dynamic factor graph (DFG) network edges identified in Brooks *et al.* (2019, Supplementary File 10). These 6863 edges are themselves considered high confidence by Brooks *et al.*, as they were selected from the network generated by DFG with time-series nitrogen-treatment response data (Marbach *et al.* 2012) based on the 71 836 validated TF–target interactions from TARGET assays. This approach of incorporating high-confidence computed interactions have been used previously to validate gene networks (Vermeirssen *et al.* 2014, Castro *et al.* 2019). The interactions from these two given networks can be considered as positives, i.e. interactions that are expected to be highly weighted edges in any predicted network. For true negatives, we used a set of 4347 interaction pairs between chloroplast-encoded and mitochondria-encoded genes (Aluru *et al.* 2022) given the unlikelihood of a direct transcriptional interaction occurring between such genes in *A. thaliana* tissues (Woodson and Chory 2008). For *A. thaliana* TF–target network reconstruction, 2015 TFs from Chen *et al.* (2017) were included.

**Table 1.** *Arabidopsis thaliana* microarray datasets.^a^

Tissue/condition	No. CEL files	No. genes
Seed	787	19 120
Flower	920	17 608
Hormone	1708	17 753
Seedling (1 week)	2822	16 298
Leaf	3564	16 887
Development	5102	18 373

a The number of CEL files and genes remaining shown are after data normalization and filtering.

All software were run on a computing cluster with each node having two 2.7 GHz 12-Core Intel Xeon Gold 6226 Processor processors and 256 GB of main memory and running RedHat Enterprise Linux (RHEL) 7.0 operating system. For the simulated and *Saccharomyces cervisiae* RNA-seq datasets, all 24 cores of a node were used for software that could be run with multiple cores. For example, Arboreto, WGCNA, TINGE, Inferelator, and MCPNet are capable of using all the cores in a node. For *A. thaliana* datasets, we used up to eight nodes and all of the 192 cores distributed across these eight nodes for Arboreto, TINGE and MCPNet, which are capable of using multiple distributed cores. While Inferelator is also capable of utilizing multiple shared cores, it was not used in this case due to extremely long run-times. Scripts used for the evaluations presented here can be accessed at https://doi.org/10.5281/zenodo.6499756.

## 4 Results

The proposed MCPNet method takes as input correlation measures, which for the purpose of evaluation presented here are the MI values between pairs of gene expression profiles. We evaluated three common methods for estimating MI values from observed data and chose adaptive partitioning (AP) with rank transform for its accuracy and speed (see Supplementary Section S1.2). We use our implementation of the AP MI estimation algorithm based on the hybrid multi-thread, multi-node parallelization approach in Section 2.3. The existing tools utilized their respective built-in MI algorithms where applicable.

The unsupervised MCP score has a single parameter, path length *L*. Empirical testing with simulated Yeast data showed that the network quality reached maximum at *L *=* *4 and improvements diminished beyond 4 (Supplementary Section S1.2). Subsequent evaluations of MCPNet, including the ensemble multipath scores, were conducted with *L* of 2, 3, and 4. The ensemble method optimizes the multipath score via a global coefficient search at a regular interval, which is a user-adjustable parameter. We used 0.1 as the search interval as it represents a good balance between search quality and computation time.

### 4.1 Evaluation using simulated yeast expression data

We begin by evaluating the effect of noise in the gene expression profile data on GCN quality. Simulated yeast datasets were generated with varying noise levels using the *NetBenchmark* package.

For each of the datasets MCPNet generates three different sets of networks: (i) *L*-path MCP score networks (i.e. MCP score matrices $R^{2},R^{3}$, and $R^{4}$ from Section 2.1); (ii) ensemble networks constructed using ensemble method discussed in Section 2.4 [i.e. $M^{4}$ matrix in Equation (4)] constructed as the weighted combination of $R^{2},\;R^{3}$, and $R^{4}$ matrices; and (iii) Stouffer-transformed ensemble network, where Stouffer’s *Z*-transform is applied to each of the $M^{4}$ matrices and the AUPRC for the best combination is reported as $M_{Z}^{4}$. This is similar to post-MI processing in CLR (Faith *et al.* 2007) to reduce background contribution to the score.

Table 2 shows the average AUPRC for networks constructed by MCPNet and other network construction methods. Results for all of the simulated data instances are given in Supplementary Table S2. The rows $R^{2},\;R^{3}$, and $R^{4}$ are networks constructed using the path scores [Equation (2)] with path lengths of 2, 3, and 4, respectively. Note that for $M^{4}$ and $M_{Z}^{4}$ networks, the average AUPRCs for the best combination is reported.

**Table 2.** Performance assessment of different gene network reconstruction methods on simulated yeast data with noise levels ranging from 0 to 1.^a^

Method	0	0.2	0.25	0.5	0.75	1.0
ARACNe-AP	0.3503	0.1829	0.1814	0.0818	0.0349	0.0170
CLR	0.1523	0.1368	0.1365	0.1193	0.1035	0.0886
MRNET	0.2711	0.2257	0.2323	0.1819	**0.1363**	**0.1075**
TINGe	0.3488	0.2478	0.2482	0.1539	0.1007	0.0666
WGCNA	0.0549	0.0545	0.0551	0.0544	0.0523	0.0496
$R^{2}$	0.3373	0.1133	0.1157	0.0615	0.0358	0.0223
$R^{3}$	0.3690	0.2575	0.2579	0.1679	0.1029	0.0619
$R^{4}$	0.3938	0.2840	0.2839	0.1959	0.1306	0.0875
$M^{4}$	**0.4198**	**0.2975**	**0.2983**	**0.2035**	0.1337	0.0889
$M_{Z}^{4}$	0.2924	0.1380	0.1399	0.1008	0.0778	0.0576
Arboreto	0.1932	0.1669	0.1637	0.1349	0.1106	0.0902
Inferelator	0.2150	0.1849	0.1763	0.1161	0.0838	0.0638

a Numbers indicate average AUPRC values over ten sampling runs. The best performing networks are highlighted in bold, and the second best is underlined. Coefficients for the best performing $M^{4}$ and $M_{Z}^{4}$ networks vary for each randomly sampled network.

As expected, the AUPRC values, and hence the network performance, decreases with increasing noise levels. Our results also show that ensemble MCP Score achieves the best performance for low and moderately noisy datasets when compared to all the other gene network reconstruction methods. At noise levels higher than 50%, MCPNet is the second best performing method. The selection of best coefficients for each of the *L*-path capacities (i.e. $R^{2},\;R^{3}$, and $R^{4}$) results in an improvement over the individual MCP score networks. By exploiting known interaction information from user supplied partial groundtruth, the ensemble approach is able to identify the best combination for each of the noise levels. Thus, MCPNet’s ensemble method is able to adjust to the unique data and noise characteristics of each dataset through fine tuning of its coefficients.

### 4.2 Evaluation with real gene expression datasets

We next evaluated performance of MCPNet using real-world data from two different organisms—yeast (*S. cervisiae*) and *A. thaliana*. For GCNs inferred from the real yeast data, our results show that Arboreto is the highest performing method followed by $M_{Z}^{4}$ (Table 3). For this dataset, all methods showed low AUPRC scores, and the absolute AUPRC differences between Arboreto and $M_{Z}^{4}$ can be considered marginal. The improvement of $M_{Z}^{4}$ over $M^{4}$ and the relatively high AUPRC for CLR suggest that Stouffer transformation as a post-processing step is beneficial for this particular dataset. While Arboreto achieved the best network quality, it does so in ∼29 h and 7 GB of memory using 24 CPU cores whereas MCPNet completed the computation in 38 s and less than half of the memory. The quality and run-time balance compared to existing tools strongly favors MCPNet for its ability to reverse-engineer a good-quality network in reasonable time. An interesting observation is the uniformly low AUPRC values when compared to those in Section 4.1, and *A. thaliana*. The same observation can be seen in Castro *et al.* (2019) which suggest this maybe inherent in the expression profile data or the groundtruth.

**Table 3.** Evaluation of MCPNet’s performance with *S. cerevisiae* data.^a^

Method	AUPRC	Time (s)		Memory (GB)		speedup
		1C	24C	1C	24C	
ARACNe-AP	0.0152	103 186	8981	81.16^c^		11.5
CLR	0.0296	1093	^d^	1.33	^d^	N/A
MRNET	0.0220	945	^d^	2.40	^d^	N/A
TINGe	0.0173	910	38	1.08	3.87	23.9
WGCNA	0.0191	116	122	3.63^c^		0.95
$R^{2}$	0.0223	401	23	1.29	2.49	17.8
$R^{3}$	0.0234	442	25	1.92	3.36	17.7
$R^{4}$	0.0240	422	25	1.78	3.22	16.9
$M^{4}$	0.0244	506	35	2.02	2.99	14.5
$M_{Z}^{4}$	0.0329	540	38	^e^	^e^	14.2
Arboreto	**0.0423**	^f^	104 662	^f^	7.14	N/A
Inferelator	0.0221	^f^	40 218	^f^	56.31	N/A

a C denotes cores.

b The best and second best scoring network by AUPRC are boldfaced and underlined, respectively. Coefficients for the best $M^{4}$ and $M_{Z}^{4}$ networks are (0.7, 0.0, 0.0, 0.3) and (0.875, 0.0, 0.0, 0.125), respectively.

c ARACNe-AP has identical memory usage for 1C and 24C, as does WGCNA.

d CLR and MRNET are not run using 24 cores as  they do not support multi-threading.

e $M^{4}$ and $M_{Z}^{4}$ are computed together in the same pass and share memory usage.

f Arboreto and Inferelator are expected to exceed run-time limit for single-core run.

*Arabidopsis thaliana* networks were constructed using data from the Development category (Table 1) containing the largest number of microarray datasets. Data were processed both on one node and eight distributed nodes for all network inference methods, with the exception of Inferelator which does not support multi-node execution and whose run-time for the development dataset is expected to exceed the cluster’s job scheduler time limit. Table 4 shows that for the *A. thaliana* dataset, $R^{4}$ and $M^{4}$ produced the best results when compared to all other methods. Importantly, all the MCPNet algorithm variants ($R^{2},\;R^{3}$, and $R^{4}$) and ensemble methods ($M^{4},\;M_{z}^{4}$) outperformed all existing network inference methods in terms of AUPRC. With the large *A. thaliana* data, MCPNet’s performance advantage over existing tools is even more evident. The two tools with the next best AUPRC values, CLR and Arboreto, required 18.5 h using 1 CPU core and 44.1 h and 437 GB of memory using eight nodes, respectively, while MCPNet completed the computation in <10 min on one node and 84 s on eight nodes with moderate per node memory footprints.

**Table 4.** Performance assessment of MCPNet using large-scale *A. thaliana* data.^a^

Method	AUPRC	Time (s)		Memory (GB)		Speedup
		1N	8N	1N	8N${}^{⋄}$	
ARACNe-AP	0.4068	1 028 618	^c^	82.41	^c^	N/A
CLR	0.4858	66 476^d^	^c^	8.94	^c^	N/A
MRNET	0.4385	25 714^d^	^c^	11.67	^c^	N/A
TINGe	0.3987	17 473	2229	4.84	19.04	7.84
WGCNA	0.4399	671	^c^	49.80	^c^	N/A
$R^{2}$	0.6208	352	51	24.08	104.77	6.90
$R^{3}$	0.6386	465	70	33.06	148.99	6.64
$R^{4}$	0.6413	431	63	31.62	147.53	6.84
$M^{4}$	**0.6414**	574	84	29.11	137.52	6.83
$M_{Z}^{4}$	0.5921	600	87	^e^	^e^	6.90
Arboreto	0.4937	^f^	158 900	^f^	437.11	N/A

a N denotes nodes.

b Coefficients for the best $M^{4}$ and $M_{Z}^{4}$ networks are (0.2,0.2,0.2,0.4) and (0.0,1.0,0.0,0.0), respectively.  Total memory usage for eight nodes is reported.

c ARACNe-AP, CLR, MRNET, and WGCNA do not support multiple nodes.

d CLR and MRNET do not support multi-threading so the experiments used only 1 core of the node.

e $M^{4}$ and $M_{Z}^{4}$ are computed together in the same pass and share memory usage.

f Arboreto is expected to exceed run-time limit for single-node run.

Finally, we evaluate the proposed methods using six *A. thaliana* datasets of different tissues and experimental conditions, each with differing number of samples and genes (see Table 1). Table 5 shows the AUPRC and run-times for the multipath ensemble MCP score network ($M^{4}$) and the next best results amongst ARACNe-AP, CLR, MRNET, TINGe, WGCNA, and Arboreto. Complete Results for all the methods are given in Supplementary Table S3. For all of the datasets analysed, MCPNet $M^{4}$ shows higher or equivalent AUPRC values while the next best AUPRC is achieved by CLR, MRNET, or Arboreto depending on the datasets, while being consistently faster by up to three orders of magnitude.

**Table 5.** AUPRC and run-time of $M_{4}$ and the next best results for networks constructed for different *A. thaliana* tissue datasets using eight 24-core nodes.

	Mil. Elem.		AUPRC		Run-time (s)	
Tissue	Input	Network	MCPNet	2nd Best	MCPNet	2nd best
Seed	15.0	365.6	0.4373	0.4285^b^	62	11 298
Flower	16.2	310.0	0.5024	0.4289^c^	52	11 830
Hormone	30.3	314.5	0.4709	0.4256^d^	52	26 798
Seedling (1 week)	46.0	265.6	0.5227	0.4424^c^	59	22 336
Leaf	60.2	285.2	0.6205	0.4529^b^	53	39 202
Development	93.7	337.6	0.6414	0.4937^d^	84	158 900

a The software that produced the next best AUPRC are noted as footnotes. The first two columns report the input and output matrix sizes as millions of elements.

b CLR.

c MRNET.

d Arboreto.

MCPNet consistently produces networks that are amongst the highest in quality as assessed by AUPRC and fastest in performance by orders of magnitude (see Supplementary Section S1.3 for a more in-depth computational performance discussion). Our results show that dataset characteristics can significantly influence the quality of the reconstructed network. The coefficients of the weighted average of *L*-path capacities for the best performing $M^{4}$ and $M_{z}^{4}$ networks also varied by dataset as noted in Tables 3 and 4. The optimization approach therefore is well-suited for the ensemble methods as it adjusts automatically to data characteristics. In addition, since MCPNet is capable of efficiently generating multiple networks in one run ($R^{2},\;R^{3},\;R^{4},\;M^{4}$, and $M_{z}^{4}$), the user can efficiently choose and further evaluate the networks with an appropriately chosen, biologically relevant downstream metric.

### 4.3 GRNs with synthetic and real yeast data sets

In this section, we evaluate MCPNet for the task of reconstructing GRNs, namely to compute the maximum path capacities between TFs and their target genes. By the definition of indirect interactions in Section 2.1, indirect TF–target interaction path may be modeled as a combinatorial sequence of TF–TF, TF–gene, and gene–gene interactions. For this evaluation, we focus on the mathematical path model consisting of a sequence of TF–TF interactions followed by one TF–target interactions as a simplification, similar to ARACNe-AP’s DPI modeling for length 2 indirect interactions. We note that an alternative simplification, TF–target gene…gene–gene, is trivially contained in the GCN output matrix.

GRNs were reconstructed MCPNet, ARACNe-AP, Arboreto, and Inferelator using real yeast and noise-free simulated datasets. CLR, MRNET, TINGe, and WGCNA were not included as they do not support GRN inferencing. *Arabidopsis thaliana* is not used for the evaluation as its true negative groundtruth do not involve the genomic TFs, thus precluding evaluation by AUPRC.

Table 6 shows the qualities of the reconstructed GCNs and GRNs. All of the reconstructed TF–target GRNs have significantly higher AUPRC than their GCN counterparts. MCPNet methods, $M^{4}$ and $M_{Z}^{4}$ particularly, outperformed existing methods for both GCN and GRN reconstructions using simulated datasets. For the real yeast dataset, MCPNet’s $M_{Z}^{4}$ method is second only to Arboreto in network quality, reflecting the same trend as for the GCN reconstruction. The high AUPRC values suggest that the mathematical formulation and algorithm implementation of MCPNet is well suited for both GCN and GRN inference.

**Table 6.** Evaluation of MCPNet’s performance in reconstructing TF–target GCN and GRN using simulated and real-world *S. cerevisiae* data on 24 cores.^a^

Method	Simulated		Real-world Yeast	
	GCN AUPRC	GRN AUPRC	GCN AUPRC	GRN AUPRC
ARACNe-AP	0.3503	0.3667	0.0152	0.0183
$R^{2}$	0.3373	0.3646	0.0223	0.0315
$R^{3}$	0.3690	0.4003	0.0234	0.0281
$R^{4}$	0.3938	0.4988	0.0240	0.0277
$M^{4}$	**0.4198**	**0.5069**	0.0244	0.0317
$M_{Z}^{4}$	0.2924	0.4545	0.0329	0.0355
Arboreto	0.1932	0.3348	**0.0423**	**0.0385**
Inferelator	0.2150	0.3413	0.0221	0.0285

a The best and second best scoring network by AUPRC are boldfaced and underlined, respectively.

### 4.4 Ensemble optimization with partial ground truths

The MCPNet ensemble method optimizes the coefficients of $\eta_{s,t}^{2},\;\eta_{s,t}^{3},\;\eta_{s,t}^{4}$ to maximize the AUPRC given available partial groundtruth and gene expression data as input. The $M^{4}$ results in Table 2 were generated by optimizing the ensemble network AUPRC using the full groundtruth. For real-world data, often only partial groundtruth is available, and the size and membership of the partial groundtruth affect network quality.

To assess the impact of the partial groundtruth, we divide the full yeast groundtruth randomly into training and testing sets, and use the training set to optimize for an ensemble network from the noise-free simulated yeast gene expression data. The ensemble network is then evaluated using the test set and the full groundtruth. As the AUPRC is parameterized with different groundtruth, effectively the AUPRC functions for optimization and final network evaluation are distinct and independent. This process is repeated 100 times to characterize the distribution of the AUPRC values and the robustness of the ensemble algorithm with varying groundtruth. We evaluated training–testing split proportions of 0.5%, 1%, 2%, 5%, and 10% of the full groundtruth. The yeast groundtruth contain 92 732 gene–gene interactions, representing 0.57% of all possible gene–gene interactions between 5716 yeast genes.

**Figure 3.**
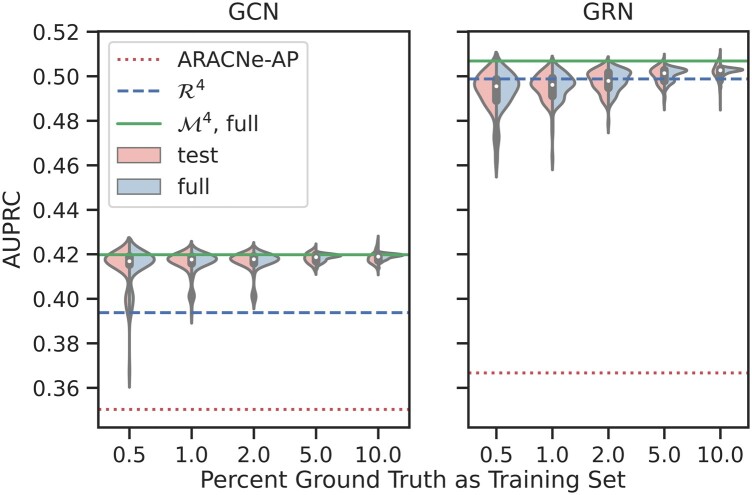
The distributions of AUPRC values for the ensemble GCNs (left) and GRNs (right) produced from training sets randomly selected from the full and TF-only groundtruth, respectively. The horizontal axes show the percentages of the full or TF-only groundtruth used as training set. Each violin plot shows the AUPRCs evaluated against the test set (left half of violin), and the full groundtruth (right half of violin). The solid green, dashed blue, and dotted red lines show the AUPRC values of the ensemble networks generated from the full or TF-only groundtruth, the $R^{4}$ network, and ARACNe-AP, respectively.

Figure 3 shows the distributions of the AUPRC values for each training–testing splits and full groundtruth. As expected, smaller training sets produced greater AUPRC value dispersion, as the ensemble network optimization relies on fewer groundtruth elements, and AUPRC computation is more influenced by variations in the gene–gene or TF–target interactions.

In all GCN cases, the AUPRC values are tight in dispersion, with standard deviations for the test AUPRCs being 0.0118, 0.0045, 0.0040, 0.0023, and 0.0026 for the 0.5–10% training–testing splits, respectively. Even for networks generated from only 0.5% of the full set, the AUPRC distribution lies completely above the ARACNe-AP’s AUPRC value of 0.3503 and nearly all above $R^{4}$’s AUPRC of 0.3938, and the majority of the networks have AUPRC more than 0.4. The means, 0.4135, 0.4169, 0.4173, 0.4184, and 0.4201, are remarkably close to the 0.4198 AUPRC of the network optimized with the full groundtruth, despite the networks being generated using small training sets. The similarity of the AUPRC distributions between test sets and full groundtruth reflects the fact that the test sets are 90–99.5% identical.

For the GRN cases, the AUPRC values are significantly higher than their GCN counterparts, likely due to better curated groundtruth. The AUPRC distribution standard deviation tightens (0.0088, 0.0063, 0.0050, 0.0036, 0.0036) as the size of training set increases. Similarly, the mean AUPRCs (0.4979, 0.4999, 0.5025, 0.5054, 0.5070) for the training–testing splits are higher than that of $R^{4}$, 0.4988, and converges to the AUPRC of the ensemble network optimized using the full TF-only groundtruth, 0.5069, even when training set represents a small fraction of the already small set of TF-only groundtruth. Regardless of the training set split, however, MCPNet’s ensemble network outperforms ARACNe-AP significantly.

These results suggest that even when the ensemble network is optimized using a small partial groundtruth set, with high probability it will be of higher quality than the $R^{4}$ network. Furthermore, a small partial groundtruth, even at 1% of full groundtruth, will on average produce a network with similar quality as one optimized using the full groundtruth. Finally, the ensemble method is robust to varying make-up of the training set, and thus the choice of ensemble weights.

## 5 Discussion

The efficient algorithm and parallel implementation of MCPNet creates opportunities for data exploration and biological hypothesis testing. Its scalability to hundreds of CPU cores enables analyses of data with sample and gene counts that are previously prohibitive, and exploration of parameter spaces and multiple methods at the same time, e.g. computing length-2, -3, and -4 MCP scores, our ensemble network and its Stouffer transform in one pass. This is useful where the individual methods are suspected to behave in a data-dependent manner, e.g. with Stouffer transform in CLR and $M_{Z}^{4}$ for the real yeast data, and inspecting multiple networks is desirable. In addition, as the MCP score formulation differs from existing algorithms, MCPNet provides complementary network that can contribute to multi-method ensemble approaches, i.e. combining networks generated by the tools evaluated in this work.

There are multiple aspects of the MCPNet algorithm and implementation that deserve further exploration. An important consideration for MCPNet development is the usability of the software. While the evaluations in this paper utilized nodes in a high performance cluster, the real yeast and the *A. thaliana* experiments showed that commodity personal computers are well suited for smaller datasets, while professional workstations should easily exceed the requirements for processing large datasets using MCPNet. At the same time, user friendly integration into common, existing bioinformatic programming environments and pipelines will ease downstream processing and biological interpretation of the reconstructed network and promote user adoption. Memory footprint reduction and language binding for Python and R/Bioconductor represent two future software engineering efforts for MCPNet.

The MCP mathematical model underlying MCPNet is agnostic of the edge weight formulation. While we focused on MI values as edge weights, other measures such as Boolean values and Pearson correlations may allow different network modeling and incorporate information such as up- and down-regulations. Our evaluations showed that MCPNet performed well with MI of RNAseq and microarray data as averaging improves signal to noise ratio, but such may not be the case for single cell transcriptomic data as MI estimation with highly sparse data may present significant challenges for MCPNet and gene network reconstruction in general, and information regarding groundtruth for individual cell types is still limited for validation. Single cell transcriptomic data, promises to allow cell-type specific GCNs and GRNs, thus MCPNet applicability to such data is of significant interest.

## 6 Conclusion

In this paper, we present a new method MCPNet for GCN and GRN reconstruction that utilizes a novel edge scoring metric, the MCP score, based on the MCP problem in network and graph analysis. MCP score characterizes indirect gene–gene and regulator–target interactions to identify significant direct interactions in an unsupervised manner and with minimal user-specified parameters. We further present an ensemble approach that uses partial network groundtruth to optimize the weighted average of MCP scores from multiple *L*-paths scores, with the objective of inferring higher quality novel interactions. The MCP score methods have been shown to generate networks with competitive or superior AUPRC scores on real-world *S. cerevisiae* and *A. athaliana* datasets, while reducing run-time by several orders of magnitude relative to existing best-in-class methods.

While we have applied MCPNet for GRN and GCN reconstruction, it operated on gene expression profile from a single time point, thus cannot infer causality, similar to the other existing tools evaluated in this work. As a consequence, the predicted gene co-expressions and regulatory interactions must be considered as candidates for further validation. With this understanding, the combination of network quality and computational performance suggests MCPNet to be a viable first-choice tool for gene network reconstruction and candidate gene and TF–target interactions.

## Supplementary Material

btad373_Supplementary_Data

## Data Availability

The data underlying this article are available in Zenodo, at https://doi.org/10.5281/zenodo.6499721. Simulated yeast datasets were generated using scripts in https://doi.org/10.5281/zenodo.6499755. Real Yeast dataset (Castro *et al.* 2019) were retrieved from https://github.com/simonsfoundation/multitask_inferelator/tree/AMuSR. *Arabidopsis thaliana* datasets were derived from NCBI SRA and the accession numbers are listed in Zenodo.
