# Supplementary material for: MCPNet: a parallel maximum capacity-based genome-scale gene network construction framework
Source: Bioinformatics. 2023 Jun 8;39(6):btad373. doi: 10.1093/bioinformatics/btad373 (PMC10287961; doi:10.1093/bioinformatics/btad373)
Supplement: btad373_Supplementary_Data [file btad373_supplementary_data.zip › BIOINF-2022-0845.accepted_supplemental.pdf]

S1 Supplementary Material

S1.1 MCP Score and Data Processing Inequality

Previous works on identification of indirect effects in the inferred gene networks are based on Data Processing Inequality (DPI), a property in information theory. In this section, we discuss how DPI-based evaluation can be modeled as a specialization of the L-path MCP score defined in section 2.1.

DPI has been adopted by ARACNe-AP and was shown to scale to tens of thousands of genes with TINGe. In these works, DPI-based algorithms compare the direct interaction (MI) to the indirect interactions through a single intermediary.

DPI. Let v_s and v_t be two genes and $v_i \in V$ be some intermediate gene through which v_s and v_t can indirectly interact, forming a Markov chain for the corresponding random variables X_s, X_i, and X_t: $X_s \rightarrow X_i \rightarrow X_t$. DPI states that for such a Markov chain, mutual information between the genes satisfy the inequalities $W_{si} \geq W_{st}$ and $W_{it} \geq W_{st}$. If both the conditions $W_{si} \geq W_{st}$ and $W_{it} \geq W_{st}$ are met, then the Markov chain is a valid information transmission path with an equal or greater capacity than the direct interaction between (X_s, X_t). Formally, this is expressed as

$$W'_{st} = \begin{cases} 0, & \begin{array}{l}\text{if } \exists X_i \text{ s.t. } W_{si} \geq (1+\tau)W_{st} \\ \quad \text{and } W_{it} \geq (1+\tau)W_{st}\end{array} \\ W_{st}, & \text{otherwise.} \end{cases} \tag{5}$$

where W' is the filtered mutual information matrix, and τ is a tolerance factor to adjust for data characteristics such as noise. In the cases where W'_{st} is 0, the direct edge (v_s, v_t) is considered superfluous and removed.

DPI related to MCP Score. The two inequalities in Equation 5 are simultaneously satisfied if the *smaller* of W_{si} and W_{it} satisfies the inequality. Furthermore, for W'_{st} to be set to zero, it suffices that the maximum of $\min(W_{si}, W_{it})$ amongst all possible intermediary genes v_i satisfies the inequality. Equation 5 can therefore be reformulated as below:

$$W'_{st} = \begin{cases} 0, & \begin{array}{l}\text{if } \max_i \left(\min(W_{si}, W_{it})\right) \\ \quad \geq (1+\tau)W_{st}\end{array} \\ W_{st}, & \text{otherwise} \end{cases} \tag{6}$$

The tolerance parameter τ in equations 5 and 6 has to be chosen manually by examining the output and optimized via trial and error. In the absence of groundtruth, choosing τ is subjective and agnostic of the data. The parameter in Equation 6 can be isolated to suggest a parameter-free gene-gene interaction score γ:

$$\begin{aligned} \gamma &= W_{st} / \max_i \left(\min(W_{si}, W_{it})\right) \\ &\leq 1/(1+\tau) \end{aligned} \tag{7}$$

The score γ is identical to $\rho^2_{st} = W_{st}/\eta^2_{st}$ from equations 2 , thus demonstrating that the 2-path MCP score is closely related to DPI.

The 2-path MCP score ρ^2_{st} can be interpreted as the relative strength of the direct interactions between genes v_s and v_t to the maximum of all possible indirect interactions between them via some gene v_i. In contrast to the DPI-based approach used in ARACNEe and TINGe, 2-path MCP score with MI is directly derived from the gene expression profile data and does not carry discontinuities introduced through a tolerance parameter. Furthermore, MCP scores for all gene pairs also enables precision-recall analyses and better gene interaction predictions based on statistically derived thresholds.

S1.2 MCPNet Parameter Selection

The MCPNet method has a few parameters: primarily the correlation method used to generate the input for the MCP scoring algorithm and the path length L. For the ensemble multipath score, there are two additional parameters: the set of path lengths considered in the ensemble, and the sampling interval during the global search for the optimal L-path capacity coefficients. The sampling interval is a user specified parameter that effects a balance between the quality of the global coefficient optimization and compute time constraints. For this paper, we chose to evaluate the method with a sampling interval of 0.1 as the additional compute time this choice incurs is acceptable.

Accurate Estimation of MI from observed data is a difficult problem and numerous methods and multiple surveys have been published (Walters-Williams and Li, 2009; Schaffernicht *et al.*, 2010; Doquire *et al.*, 2012). While a straightforward method of binning observations and empirically computing MI can be employed (such as by MRNET), the B-spline (Daub *et al.*, 2004) and adaptive partitioning (AP; Seok and Kang (2015)) methods are two of the most commonly applied methods in GRN inference. Previous studies also show that these two methods are superior to the binning method(s) in estimating MI Daub *et al.* (2004); Liang and Wang (2008). B-spline method, first introduced as an alternative to binning in the context of clustering of gene expression profiles, has been used in many GRN methods including CLR, ARACNe and TINGe. ARACNe-AP (Lachmann *et al.*, 2016), the latest update to the ARACNe implementation, uses the AP method for computing MI.

Using simulated yeast data, we first evaluated run-time and accuracy of different MI estimation methods, including our own implementations of B-spline and AP. For B-spline implementations, the typical B-spline parameters of ten (10) bins and three (3) degrees of freedom were employed based on prior experience (Hartemink, 2005; Faith *et al.*, 2007; Aluru *et al.*, 2013). AP parameters were discussed in detail in Lachmann *et al.* (2016). Table S1 shows that AP with rank transformed gene expression data as adopted by ARACNe-AP and MCPNet performed better than B-spline based methods with significantly higher AUPRC values. AP also has a computational advantage over B-spline based methods. Our implementation based on the same strategy as in Section 2.3 demonstrated superior computational performance for AP when compared to all other B-spline algorithms. Therefore, subsequent studies were all conducted using AP with rank transformation.

We next examined the effect of increasing path length L on the quality of the inferred GRN from the simulated Yeast dataset. For each path length L between 2 and 30, the L-path MCP score is calculated as a score matrix, which is then evaluated against the groundtruth network to calculate the AUPRC. We observed that AUPRC of the MCP scores increases to its maximum rapidly for small L, then reduces slightly and settles into a

Table S1: Evaluation of two different MI implementations on simulated yeast data.

Method	Implementation	AUPRC	Runtime (s) 1C	24C
B-Spline	CLR	0.0671	620	∅
B-Spline + RT	TINGe	0.0922	84	6
AP + RT	ARACNe-AP	0.2020	3481	∅
Binning w. Pearson Estimator	MRNET	0.0648	14	∅
B-Spline	MCPNet	0.0672	63	3
AP + RT	MCPNet	0.2021	20	1

Note: AP denotes adaptive partitioning, and RT indicates rank transform prior to MI computation. ∅CLR and ARACNe-AP do not have the capability to compute MI on multiple shared cores.

steady oscillation (data not shown). For the simulated yeast dataset and adaptive partitioning MI, the peaks occurs at $L = 4$, and stabilization occurs at $L = 9$. Subsequent evaluations of both MCP scores and the multipath ensemble score employ $L \leq 4$ as a reasonable balance between computational cost and quality improvement.

S1.3 Computational Performance of MCPNet

In addition to network quality, Tables 3, 4, and 5 report the run-times, memory utilization, and parallel speed up of the proposed and existing methods. The reported times are for the full processing pipelines including file input and output.

Evaluation with the synthetic yeast dataset used a single compute core and 24 shared-memory cores in multi-threaded configuration. Arboreto and Inferelator were run only as multi-threaded as the extrapolated single-core run-time is above job scheduler limit. Table 3 shows that ARACNe-AP, Arboreto and Inferelator respectively required 2.38, 29.07 and 11.17 hours to construct the network using 24-cores, and ARACNe-AP used approximately 29 hours on 1 core to do the same. These represent the worst performing methods.

Of the remaining methods, MCPNet demonstrated the highest absolute computational performance on 24-cores, and on single-core is only bested by WGCNA, which is based on the simpler Pearson correlation and has lower AUPRC score than MCPNet. MCPNet is approximately $2\times$ faster than CLR, MRNET, and TINGe using a single core. On 24 cores, TINGe has similar or worse run-time than MCPNet, while CLR and MRNET do not support multi-threading. Interestingly, even though WGCNA supports multi-threading, in practice it experienced a slow-down.

From a parallel scaling perspective, TINGe achieves a nearly perfect speed up of $24\times$ when core count increased from 1 to 24, while our proposed methods obtained speed ups of approximately $17\times$ to $18\times$. MCPNet's lower speedups are likely due to the remaining non-parallelizable steps, such as file input and output, occupying a greater proportion of the total run-time.

MCPNet's performance advantage over the existing methods is more apparent when processing large datasets and in high performance computing environments. Table 4 shows that with one 24-core node, MCPNet outperforms all existing tools – CLR, MRNET, and TINGe required 18.5, 7.1, and 4.9 hours to complete, while at the extreme ARACNe-AP required 285.7 hours. In contrast, MCPNet required between 6 to 10 minutes. The fastest MCPNet method is nearly $3,000\times$ faster than ARACNe-AP, while the most costly MCPNet method is still more than $1,700\times$ faster.

The performance advantage of MCPNet is equally striking when 8 nodes (192 cores) are used. Only MCPNet, TINGe, and Arboreto are capable of utilizing multiple nodes. MCPNet significantly outperforms both, up to $43.7\times$ faster than TINGe and $3,115\times$ faster than Arboreto, when compared using $\mathcal{R}^2$. The absolute run-times for MCPNet methods ranges between 1 to 1.5 minutes. Compared to the single-node ARACNe-AP run-time, $\mathcal{R}^2$ is $20,169\times$ faster. Run-time at this scale renders feasible parameter studies with large gene expression datasets. We also observed parallel scaling factor between 6.64 and 6.90 for MCPNet when node count increased from 1 to 8, while TINGe achieved a closer-to-perfect scaling factor of 7.84 out of 8. TINGe's longer run-time may be attributed to its higher network communication reliance, while MCPNet's lower scaling is due to the larger portion of the remaining, unparallelizable components in the pipeline.

Table 5 further illustrates the performance advantage of MCPNet. For the six datasets with varying sample and gene counts, the $\mathcal{M}^4$ method, the second most computationally intensive MCP score-based methods, is consistently faster by three to four orders of magnitude when compared to the method that produced the next best network as per AUPRC. The $(O(|S||V|^2))$ MI computation complexity and the $(O(|V|^3 \log_2(L))$ MCP score complexity imply that the run-time of MCPNet scales well with both input sample and gene counts during the MI computation, and with the network size during the L-path capacity and score computation.

The memory consumption of MCPNet implementations is comparable to that of existing tools including CLR, MRNET, TINGe, WGCNA, and Arboreto for the yeast dataset, both in single thread and multi-thread modes (Table 3). In contrast, ARACNe-AP and Inferelator required significantly more memory. For small networks, MCPNet is suitable even on laptop and desktop class systems. For the *A. thaliana* dataset (Table 4, when executing on a single node, the memory consumption is comparable to WGCNA, but higher than CLR and MRNET. In the 8-node configuration, MCPNet required approximately 13GB to 19GB of memory per node due to replication of the data structures to reduce communication costs. In contrast TINGe required approximately 19GB in total, but is significantly slower due to higher communications load. The memory footprint per node is well within the specification of typical workstation and server class computers. While MCPNet does not present the lowest memory footprint, it balances memory consumption to achieve low communication and high computational performance.

Our evaluations indicate that while dataset characteristics play an important role, the proposed MCPNet methods consistently produce networks that are amongst the highest quality ones based on the AUPRC metric. The methods are among the fastest alongside TINGe and WGCNA for smaller datasets on multi-core shared memory machine, and the definitively fastest methods by orders of magnitude for large datasets and in multi-node settings. While MCPNet demonstrate significant speed advantage, the existing tools may not be fully optimized for modern hardware or programming languages, and may rely on algorithms that have high computational complexity or are hard to optimize and parallelize. The combination of network quality and computational performance suggests MCPNet to be a viable first-choice tool for gene network reconstruction.

SI References

Aluru, M. *et al.* (2013). Reverse engineering and analysis of large genome-scale gene networks. *Nucleic Acids Research*, **41**(1), e24–e24.

Daub, C. O. *et al.* (2004). Estimating mutual information using b-spline functions–an improved similarity measure for analysing gene expression data. *BMC Bioinformatics*, **5**(1), 1–12.

Doquire, G. *et al.* (2012). A comparison of multivariate mutual information estimators for feature selection. In *ICPRAM (1)*, pages 176–185.

Faith, J. J. *et al.* (2007). Large-scale mapping and validation of Escherichia coli transcriptional regulation from a compendium of expression profiles. *PLoS Biology*, **5**(1), e8.

Hartemink, A. J. (2005). Reverse engineering gene regulatory networks. *Nature Biotechnology*, **23**(5), 554–555.

Lachmann, A. *et al.* (2016). ARACNe-AP: gene network reverse engineering through adaptive partitioning inference of mutual information. *Bioinformatics*, **32**(14), 2233–2235.

Liang, K.-C. and Wang, X. (2008). Gene regulatory network reconstruction using conditional mutual information. *EURASIP Journal on Bioinformatics and Systems Biology*, **2008**, 1–14.

Schaffernicht, E. *et al.* (2010). On estimating mutual information for feature selection. In *International Conference on Artificial Neural Networks*, pages 362–367. Springer.

Seok, J. and Kang, Y. S. (2015). Mutual information between discrete variables with many categories using recursive adaptive partitioning. *Scientific Reports*, **5**(1), 1–10.

Walters-Williams, J. and Li, Y. (2009). Estimation of mutual information: A survey. In *International Conference on Rough Sets and Knowledge Technology*, pages 389–396. Springer.
